# Prevalence of intestinal parasites and associated risk factors among inmates at correctional facilities in Central Zambia

**DOI:** 10.1016/j.parepi.2026.e00503

**Published:** 2026-04-01

**Authors:** Gilbert Munsaka, Jonathan Gwasupika, Ruth L. Mfune, Imukusi Mutanekelwa, Bupe Chewe, Clyde M. Hakayuwa, Mabvuto Daka, Mwape Chipampe, Maisa Kasanga, Andrew M. Phiri, Mable Mutengo, Oliver Mwenechanya, Steward Mudenda, Victor Daka

**Affiliations:** a University Teaching Hospital, Department of Pathology and Microbiology, Lusaka, Zambia; b National Health Research and Training Institute, Clinical Sciences Department, Ndola, Zambia; c Copperbelt University, Michael Chilufya Sata School of Medicine, Department of Public Health, Ndola, Zambia; d Zambia Correctional Services, Medium Prison Health Post, Kabwe, Zambia; e University of Zambia, School of Veterinary Medicine, Clinical Sciences Department, Lusaka, Zambia; f University of Zambia, School of Health Sciences, Department of Pharmacy, Lusaka, Zambia; g Institute of Basic and Biomedical Sciences, Levy Mwanawasa Medical University, Lusaka, Zambia

**Keywords:** Hygiene, Inmates, Intestinal parasitic infections, Prevalence, Risk factors, Soil-transmitted helminths

## Abstract

**Background:**

Intestinal parasitic infections (IPIs) such as soil-transmitted helminths remain a public health concern, especially in settings with inadequate sanitation. Vulnerable groups such as prisoners, face a heightened risk due to poor hygiene, overcrowding, and limited access to healthcare. Despite this, little is known about the prevalence and risk factors for IPIs among inmates in Zambian prisons. This study aimed to investigate the prevalence of intestinal parasites in four correctional facilities in Kabwe, Central Province of Zambia.

**Methods:**

This was a cross-sectional study conducted among 260 inmates across four correctional facilities in Kabwe, Zambia. Stool samples were collected and examined using direct mount and formol-ether concentration techniques. A structured questionnaire was used to assess demographic data and hygiene practices. Data were analysed using SPSS version 25.0. Chi-square or Fisher's exact tests were used to assess associations, while multivariate logistic regression identified predictors of infection. A *p*-value <0.05 was considered statistically significant.

**Results:**

The overall prevalence of intestinal parasite infestation was 28.9%. The most common parasites identified were *Entamoeba coli* (57.3%), *Entamoeba histolytica* (24.0%), *Giardia lamblia* (9.3%), hookworms (8.0%), and *Ascaris lumbricoides* (1.4%). Univariate analysis revealed significant associations between parasite infestation and soap use (*p* < 0.0001) and prison classification (*p* = 0.023). In the multivariate model, soap use remained significantly protective. Inmates who used soap were 84% less likely to have parasitic infestation compared to those who did not (AOR = 0.16, 95% CI: 0.07–0.40, p < 0.0001).

**Conclusion:**

The study revealed a high burden of intestinal parasitic infestations among inmates, with limited hygiene practices, such as a lack of soap use, being a significant contributor. Interventions to improve access to soap and enhance hygiene education within correctional facilities are urgently needed to reduce the burden of parasitic infections.

## 1 Introduction

Intestinal parasitic infections (IPIs) continue to be a global public health concern, with soil-transmitted helminths (STHs) listed among the most prevalent (Ahmed, 2023). The World Health Organization (WHO) estimates that approximately 1.5 billion individuals which represents 24% of the global population; are infected with STHs in Africa; Asia; and Latin America (Ahmed, 2023; Rahimi et al., 2023; WHO, 2023). *Strongyloides stercoralis* infections alone affect more than 600 million people worldwide, especially in regions with inadequate sanitation, where its distribution overlaps with other STHs (Pillai et al., 2024). Annually; about 10.5 million new cases of IPIs are reported (Mussema et al., 2024). A recent systematic review reported an overall prevalence of intestinal parasitic infections of 34.0% among institutionalized populations. Within this group; prison inmates showed the prevalence of 29.7%. *Blastocystis hominis* was identified as the most common protozoan parasite; with a pooled prevalence of 18.6% (Abaka-Yawson et al., 2025). These infections disproportionately affect vulnerable populations due to limited healthcare access; poor nutrition; poverty; inadequate hygiene; and poor access to clean water and sanitation facilities (Mussema et al., 2024; Pillai et al., 2024; Wakid, 2020; WHO, 2023).

Previous studies also identified such risk factors for IPIs as educational level, age, weight, and upper arm circumference, all of which have been found to significantly correlate with infection prevalence (Khan et al., 2022). Studies also found that poor hygiene practices; consumption of contaminated water; and limited access to healthcare services contribute to the high burden of these infections. Beyond their immediate health effects; IPIs can result in chronic complications such as malnutrition; anaemia; intestinal obstruction; reduced growth rate and impaired cognitive development in children (Feleke et al., 2021; Khan et al., 2022; Prüss-Ustün et al., 2019).

Preventive measures against intestinal parasitic infections like proper hygiene practices such as avoiding soil ingestion, using latrines, and washing vegetables with clean water before consumption (Narkkul et al., 2025). Wearing protective footwear can significantly reduce the risk of hookworm infection (Strunz et al., 2014). While much of the research on IPIs has focused on children; the elderly; immigrant populations; and immunosuppressed individuals; prisoners represent a marginalized group with high susceptibility to these infections. Correctional facility inmates in developing countries often endure inadequate living conditions; malnutrition; lack of potable water; overcrowding; and poor hygiene; all of which increase their risk of acquiring parasitic infections (Jiraamonninit et al., 2006; Muñoz-Antoli et al., 2023).

The prevalence of intestinal parasites among inmate populations varies widely. Studies have reported a prevalence of 8.9% of pathogenic parasites among inmates in Spain, comparable to 8.7% of non-pathogenic species (Muñoz-Antoli et al., 2023). Higher prevalence rates have been observed in Guinea (33.5%) (Kadio et al., 2021) and Ghana (38.2%) (Ameya et al., 2019). In Ethiopia; other studies have documented the prevalence of intestinal parasites among inmates: 48.1% (Ameya et al., 2019); 39.2% (Mussema et al., 2024) and 35.6% (Nigus et al., 2025). *Entamoeba histolytica/dispar* was the most commonly detected parasite; followed by *Giardia lamblia*; *Ascaris lumbricoides*; hookworms; *Taenia* species; and *Schistosoma mansoni* (Mussema et al., 2024).

According to the World Prison Brief, Zambia's correctional facility houses approximately 25,372 inmates, despite a designed holding capacity of only 10,650 (Tembo, 2024); resulting in an overcrowding rate of 238.2%. While studies have reported a 19.6% prevalence of intestinal parasites among vulnerable populations such as children in Zambia; schistosomiasis remains one of the most endemic parasitic infections in the country (Kalinda et al., 2018). However; no study has specifically examined intestinal parasitic infections among inmates. Understanding the prevalence and associated risk factors of intestinal parasitic infections among prisoners is crucial for implementing effective interventions. If WHO is to achieve its six global objectives for helminthiasis eradication (WHO, 2023), assessing the burden of these infections across all high-risk groups is essential.

Despite the evident public health implications, there remains a paucity of research investigating the prevalence of IPIs in Zambian prisons. This study aimed to address this gap by determining the prevalence of intestinal parasitic infections and identifying associated risk factors among inmates at the Mukobeko group of correctional facilities in Kabwe, Central Zambia.

## 2 Materials and methods

### 2.1 Study site and study design

This was a cross-sectional study aimed at investigating the prevalence of intestinal parasites and associated risk factors among inmates at correctional facilities in Kabwe, Zambia from August to December 2024. Kabwe is the provincial capital of the Central Province of Zambia and hosts the largest group of correctional facilities, including two maximum security prisons (Shalala, 2020). The study was conducted at four correctional facilities located in Kabwe, including Mukobeko Maximum, Kabwe Medium, Kabwe Female Maximum and Kalonga correctional facility. The facilities comprised one medium-security and two maximum-security correctional institution.

### 2.2 Sample size estimation and sampling

The sample size was determined using Cochran's formula (Ahmed, 2024). Due to the absence of prior studies in Zambia, a conservative prevalence estimate of 50% was assumed, with a precision of 5% and a finite population of 2755 inmates across the four correctional facilities. The finite population-corrected minimum sample size was calculated to be 260. To account for potential non-response and incomplete data, the target sample size was increased to 270 participants. A total of 270 inmates were approached and recruited consecutively; however, only 260 provided complete data and stool samples suitable for analysis and were therefore included in the final analysis.

### 2.3 Sample collection and questionnaire administration

All study participants received two stool containers and were instructed to provide early morning stool samples on two consecutive days. Participants were advised to collect the samples without urine or water contamination, securely close the containers, and return them to the study team on the same day of collection. The collected samples were transported to the laboratory where they were received by the parasitology section of the Medical Laboratory Sciences Department where microscopic examination was performed by two trained laboratory experts. Any discrepancies in findings were resolved through review by a third senior laboratory scientist. In addition, a structured questionnaire was administered to gather data on participants' demographic characteristics, hygiene practices, and duration of incarceration. The questionnaire underwent expert review by four faculty members from the Copperbelt University School of Medicine and was pretested among 20 inmates at the Peter Singogo Correctional Facility in Ndola. Feedback from the pilot was used to refine the questionnaire, and the pilot data were excluded from the final analysis.

### 2.4 Laboratory analysis

Macroscopic examination of all stool samples was performed to assess the appearance and the presence of adult worms prior to microscopic analysis. Stool microscopic analysis was then conducted using two methods: the direct wet mount and the formol–ether concentration technique (Al-Refai and Wakid, 2024). For direct wet mount examination; two preparations were made on a clean microscope glass slide using 0.9% *w*/*v* normal saline and Lugol's iodine. A drop of normal saline was placed on the left side of the slide and a drop of Lugol's iodine on the right side. A small amount of stool 2 mg was emulsified separately into each drop using a sterile applicator stick. Each preparation was covered with a coverslip and examined under a light microscope at low (10×) and high magnification (40×) for intestinal parasites(Agarwal et al., 2025; Zaman et al., 2018).

For the formol–ether sedimentation technique, approximately 1 g of stool was emulsified in 10 mL of 10% formalin saline and filtered through cotton gauze into a centrifuge tube. Subsequently, 3 mL of diethyl ether was added, and the mixture was shaken vigorously for about 30 s and centrifuged at 2000 rpm for 5 min. After centrifugation, four layers were formed: a top ether layer, a debris plug, a formalin layer, and a sediment at the bottom. The supernatant was carefully discarded, and the sediment containing the diagnostic stages of parasites was examined microscopically under low and high magnification (Agarwal et al., 2025; Allen and Ridley, 1970; Demeke et al., 2021; Garba et al., 2023). Each sample was independently examined by two trained laboratory technologists, and discrepancies were resolved by a third reader (Speich et al., 2015).

### 2.5 Data analysis

Data were entered and analysed using Statistical Package for Social Sciences (SPSS) 25.0 (SPSS Inc., Chicago, II, USA). The association between duration of incarceration and intestinal parasitic infections was analysed using the Pearson chi-square test or Fischer's Exact Test, depending on which was appropriate. Predictors of intestinal infection were investigated using multivariate binary logistic regression, reporting odds ratios and their 95% confidence intervals. A *p*-value less than 0.05 was considered to be statistically significant.

### 2.6 Ethics statement

Ethical clearance was obtained from the Levy Mwanawasa Medical University Research Ethics Committee (approval number: REF No LMMU-REC 0000464/24) (Supplementary File 1). Permission to conduct the study at the Mukobeko group of correctional facilities was obtained from the Commissioner General, Zambia Correctional Service (ZSC) headquarters. Formal written informed consent was administered to all inmates, and only those who consented were included in the study. All study data were restricted to the investigators, and no personal identifiers were used as data were being entered and analysed. All inmates who were found to be positive for intestinal parasites were referred to the nearest health facilities for standard care and management.

## 3 Results

### 3.1 Socio-demographic, behavioural, and clinical characteristics of inmates by parasite infection status

The majority of inmates were male (223, 85.8%) compared with females (37, 14.5%), with most males found in both the non-infected (157, 84.9%) and infected (66, 88.0%) groups (*p* = 0.512). Most participants were aged 31–50 years (125, 48.1%), followed by those aged 15–30 years (101, 38.8%), with the highest proportions in both infection categories occurring in the 31–50-year age group (*p* = 0.635).

Most inmates had primary education (170, 65.4%), which also comprised the majority among the non-infected (118, 63.8%) and infected (52, 69.3%) groups (*p* = 0.380). A higher proportion of inmates resided in urban areas (138, 53.1%) than rural areas, with urban residents forming the majority in both non-infected (100, 54.0%) and infected (38, 50.7%) groups (*p* = 0.620).

In terms of duration of imprisonment, most inmates had been in prison for ≤2 years (110, 42.3%), and this category had the majority among infected inmates (37, 49.3%), while the non-infected group was similarly dominated by those with ≤2 years of being in prison (73, 39.5%) (*p* = 0.358). The majority of inmates did not use soap (190, 73.1%), and this group accounted for most infected cases (69, 92.0%) and non-infected cases (121, 65.4%) (***p* < 0.001**).

More inmates were in the maximum-security prisons (143, 55.0%) than medium security prisons; however, the majority of infected inmates were from medium security prisons (42, 56.0%), while non-infected inmates were mostly from maximum security prisons (110, 59.5%) (***p* = 0.023**).

Most inmates had poor hygiene status (202, 77.7%), which predominated in both the non-infected (140, 75.7%) and infected (62, 82.7%) groups (*p* = 0.220). The majority of inmates did not engage in gardening activities (159, 61.2%), forming the largest proportion in both the non-infected (114, 61.6%) and infected (45, 60.0%) groups (*p* = 0.808).

More than half of the inmates reported having knowledge of parasites (141, 54.2%), and this group constituted the majority among infected inmates (44, 58.7%), while those without parasite knowledge predominated slightly among the non-infected group (88, 47.6%) (*p* = 0.361).

Most inmates reported no diarrhoea (172, 66.1%), and this group formed the majority in both the non-infected (122, 66.0%) and infected (50, 66.7%) categories (*p* = 0.911). Similarly, most inmates reported no abdominal pain (154, 59.2%), accounting for the majority of both non-infected (108, 58.4%) and infected (46, 61.3%) inmates (*p* = 0.660).

Details in Table 1**.**

**Table 1:** Socio-demographic, behavioural, and clinical characteristics of inmates by parasite infection status.

Variable	Total, n (%)	Not infected, n (%)	Infected, n (%)	p-value
**Sex**				0.512
Male	223 (85.8)	157 (84.9)	66 (88.0)	
Female	37 (14.2)	28 (15.1)	9 (12.0)	
**Age (years)**				0.635
15–30	101 (38.8)	69 (37.3)	32 (42.7)	
31–50	125 (48.1)	90 (48.7)	35 (46.7)	
>50	34 (13.1)	26 (14.0)	8 (10.6)	
**Education level**				0.380ᶠ
Primary	170 (65.4)	118 (63.8)	52 (69.3)	
Secondary	80 (30.8)	61 (33.0)	19 (25.3)	
Tertiary	10 (3.8)	6 (3.2)	4 (5.3)	
**Residence**				0.620
Rural	122 (46.9)	85 (46.0)	37 (49.3)	
Urban	138 (53.1)	100 (54.0)	38 (50.7)	
**Duration of stay (years)**				0.358ᶠ
≤2	110 (42.3)	73 (39.5)	37 (49.3)	
3–5	95 (36.5)	68 (36.8)	27 (36.0)	
6–10	37 (14.2)	30 (16.2)	7 (9.3)	
>10	18 (6.9)	14 (7.6)	4 (5.3)	
**Use of soap**				**<0.001**^**⁎**^
No	190 (73.1)	121 (65.4)	69 (92.0)	
Yes	70 (26.9)	64 (34.6)	6 (8.0)	
**Class of prison**				**0.023**^**⁎**^
Medium security	117 (45.0)	75 (40.5)	42 (56.0)	
Maximum security	143 (55.0)	110 (59.5)	33 (44.0)	
**Hygiene**				0.220
No	202 (77.7)	140 (75.7)	62 (82.7)	
Yes	58 (22.3)	45 (24.3)	13 (17.3)	
**Gardening**				0.808
No	159 (61.2)	114 (61.6)	45 (60.0)	
Yes	101 (38.8)	71 (38.4)	30 (40.0)	
**Parasite knowledge**				0.361
No	119 (45.8)	88 (47.6)	31 (41.3)	
Yes	141 (54.2)	97 (52.4)	44 (58.7)	
**Diarrhoea**				0.911
No	172 (66.2)	122 (66.0)	50 (66.7)	
Yes	88 (33.8)	63 (34.0)	25 (33.3)	
**Abdominal pain**				0.660
No	154 (59.2)	108 (58.4)	46 (61.3)	
Yes	106 (40.8)	77 (41.6)	29 (38.7)	

Footnotes: ᴾ = p-value (Pearson's chi-square test); ᶠ = Fisher's exact test; ^⁎^ = Statistically significant.

Percentages are column percentages and may not total 100% due to rounding.

### 3.2 Prevalence of parasite infestation among inmates

After microscopic examination of the stool samples, the prevalence of parasite infestation among inmates was 28.9% (75/260) (Table 1). The most commonly isolated parasites were *E. coli* (57.3%) and *E. histolytica* (24.0%), *G. lamblia* 9.3% and *Hook worms* 8.0%.

### 3.3 Factors associated with parasite infestation among prisoners

Using multivariate binomial regression, the final model was significant (*p* < 0.05) in explaining the factors contributing to parasite infestation in prisons. The inmates who used soap were 84% (AOR 0.16, p < 0.0001, 95% CI 0.07–0.40) less likely to present with parasite infestation than those who did not **(**Table 2).

**Table 2:** Factors associated with the presence of parasites among prisoners.

Variable	Category	cOR	P-value	95% CI	aOR	P-value	95% CI
Use of Soap	No	1	–	–	1	–	–
	Yes	0.16	**0.000**	0.07–0.40	0.16	**0.000**	0.07–0.40
Hygiene	No	1	–	–	1	–	–
	Yes	0.65	0.222	0.33–1.29	0.58	0.130	0.28–1.18
Parasite Knowledge	No	1	–	–	1	–	–
	Yes	1.29	0.361	0.75–2.22	1.48	0.177	0.84–2.63
Prison Class	Medium-security	1	–	–	1	–	–
	Maximum-security	0.54	0.024	0.31–0.92	0.68	0.189	0.38–1.21

Footnotes: COR = Crude Odds Ratio; AOR = Adjusted Odds Ratio.

## 4 Discussion

This study aimed to determine the prevalence of intestinal parasitic infections and identify associated risk factors among inmates at Mukobeko Maximum Security Prison in Kabwe, Zambia. To our knowledge, this is the first study in Zambia to investigate parasites among inmates.

The findings revealed a relatively high prevalence of parasitic infections at 28.9%, with *E. coli*, *E. histolytica*, and *G. lamblia* being the most commonly identified organisms. These results are comparable to findings from previous studies, though variations in prevalence were observed across different settings. The prevalence reported in this study (28.9%) was lower than that found in Ethiopia: 42.6% in Mekelle Prison (Mardu et al., 2019) and 39.2% in Hosanna Town Prison (Mussema et al., 2024); 33.5% in Guinea (Kadio et al., 2021). Similarly; a study conducted in Ghana reported a prevalence of 38.2% (Abaka-Yawson et al., 2024); and a systematic review revealed a pooled prevalence of 43.68% (Feleke et al., 2021). However; the current findings were higher than those reported in several other countries: 15.7% in Spain (Muñoz-Antoli et al., 2023); 24.7% in Kenya (Rop et al., 2016); 22.8% in Nigeria (Nadabo et al., 2020); 6% in Nepal (Shrestha et al., 2019); 7.89% in India (Amit et al., 2016); 26.5% in Malaysia (Angal et al., 2015); and 20.2% in Brazil (Curval et al., 2017). These differences in prevalence rates may be as a result of variations in environmental sanitation, access to clean water, personal hygiene practices, deworming programs, diagnostic techniques used, prison overcrowding, and the general health infrastructure of correctional facilities in different countries.

Although most inmates in our study reported good hygiene knowledge and practices, only 26.9% used soap after using the toilet. Notably, multivariate analysis showed that soap use had a significant protective effect, with inmates who used soap being 84% less likely to be infected.

In terms of the highest reported organism, the most commonly reported intestinal parasites in this study were *E. coli* and *E. histolytica/dispar*, which is consistent with findings from some sub-Saharan African countries. For instance, in Ethiopia, *E. histolytica/dispar* was found in 23.3% of samples (Mardu et al., 2019). Similarly; a prison-based study by Mussema in the same country also reported *E. histolytica/dispar* and *G. lamblia* as the leading parasites; contributing to the overall prevalence of 39.2% (Mussema et al., 2024). In Ghana; *E. histolytica* was again the dominant parasite; which affirms its regional predominance (Abaka-Yawson et al., 2024).

However, notable geographic variations in parasite profiles were observed in other regions. In Brazil, for example, the most prevalent parasite was *B. hominis*, found in 28.2% of participants, followed by *Endolimax nana* (14.9%), *G. duodenalis* (11.0%) and *A. lumbricoides* (5.0%) (Seguí et al., 2018). In contrast; studies in Malaysia reported *S. stercoralis* as the most common; with a high prevalence of 15.8% among schoolchildren (Al-Mekhlafi et al., 2019). Kenya's Nyanchongi study also revealed high rates of *Trichuris trichiura* (28.3%) (Kepha et al., 2024); and in urban Malaysia; *T. trichiura* dominated with a 64.2% prevalence (Chin et al., 2016); while in another study within the country was *A. lumbricoides* and *Clonorchis sinensis* were less frequent at 0.8% each (Jamaiah and Rohela, 2005).

These variations can be explained by differences in environmental and climatic conditions, hygiene practices, and parasite ecology. *E. coli, E. histolytica/dispar* and *G. lamblia* thrive in areas with poor water sanitation, which is common in many African prisons, facilitating faecal-oral transmission (Hailu and Alemu, 2024). Although *E. coli* is a non-pathogenic protozoan; its presence is generally regarded as an indicator of faecal contamination and poor hygiene (Haidar and Jesus, 2023). This suggests that the study population may be routinely exposed to contaminated water; food; or surfaces; likely due to inadequate sanitation and hygiene practices. In contrast; the high prevalence of *S. stercoralis* in tropical Malaysia may be due to the parasite's ability to penetrate the skin in moist; humid environments—conditions that are less common in drier African climates. Soil-transmitted helminths like *A. lumbricoides* and *T. trichiura* require specific soil and temperature conditions for their eggs to mature and become infective (Amor et al., 2016; Scarso et al., 2024). Hence, they are more prevalent in regions with frequent rainfall and loose, moist soil, such as in parts of Brazil and Southeast Asia.

Differences in host behaviors and socioeconomic factors also contribute to the variation. Marginalized populations, such as the prison populations in Ethiopia, often experience overcrowding and limited access to clean water or healthcare, increasing the risk of infection (Engdaw et al., 2023). Periodic mass deworming and improved public health interventions have been successfully implemented in countries like Kenya (AMREF, 2023), which may help explain the relatively lower prevalence of *Entamoeba* and *Giardia* species there.

This study also identified risk factors associated with intestinal parasitic infections among inmates. The study observed that the use of soap after toilet use emerged as a protective factor. This finding aligns with research from Ethiopia (Mussema et al., 2024); where inmates not using soap after toilet use had a higher likelihood of infection. Soap reduces the surface tension of water and emulsifies fats and oils on the skin; which helps physically remove dirt; and faeces; and washing away the parasitic infective stages; leading to interruption of the life cycle of these organisms (Alqarni et al., 2023)**.**

These findings highlight the importance of promoting proper hygiene practices as washing hands, ensuring access to sanitation facilities, and addressing environmental conditions within prisons to reduce the prevalence of intestinal parasitic infections among inmates. They also have public health implications such as increased incidence of parasitic organisms.

The high prevalence of intestinal parasitic infections in this study, especially soil-transmitted helminths, reinforces the urgent need to devise targeted public health interventions such as scaling up mass drug administration, improving access to clean water, sanitation, and hygiene, and strengthening health education, especially in high-risk environments such as correctional facilities. According to WHO, the global target is to eliminate STH as a public health problem by 2030 by reducing the prevalence and associated morbidity (World Health Organization, 2020). These efforts align with Sustainable Development Goals (SDGs) like SDG 3 (Good Health and Well-being) by reducing disease burden, SDG 6 (Clean Water and Sanitation) by preventing faecal-oral transmission, SDG 1 (No Poverty), and SDG 10 (Reduced Inequalities) by addressing health disparities that disproportionately affect vulnerable populations like prisoners.

### 4.1 Limitations

The cross-sectional design limited the causal inference between identified risk factors and intestinal parasitic infections; hence only associations were determined. The study was conducted in a single group of correctional facilities in one location (Kabwe), which may limit the generalizability of the findings to other prisons in Zambia. Hygiene practices and symptoms were self-reported and therefore subject to recall and social desirability bias, and environmental factors such as water quality, sanitation conditions, and prior deworming history were not assessed.

Moreover, we did not perform special staining or molecular testing for coccidian protozoa, including *Cryptosporidium*, *Isospora*, and *Cyclospora*, as well as *Microsporidia*. These opportunistic enteric pathogens are important in immunocompromised people and may contribute significantly to gastrointestinal infections. This may have resulted in under-detection of these organisms, thereby underestimating their true prevalence. Future studies incorporating targeted diagnostic approaches would provide a more comprehensive understanding of the burden of parasitic infections at this institution. Despite these limitations, this study brings up very important insights that will guide the management and control of parasitic infections.

## 5 Conclusion

The study found a relatively high prevalence of parasitic infections among inmates, with *E. coli, E. histolytica and G. lamblia* being the most common parasite isolated. A significant association was observed between parasite infestation and poor hygiene practices, especially the lack of soap use after toilet visits. Despite high levels of awareness about hygiene, consistent practice remains low.

## Consent to publish declaration

This manuscript does not contain any individual person's data (including individual details, images, or videos). Therefore, Consent to Publish is not applicable.

## Ethics and consent to participate declarations

Ethical clearance was obtained from the Levy Mwanawasa Medical University Research Ethics Committee (approval number: REF No LMMU-REC 0000464/24) (Supplementary File 1). Permission to conduct the study at the Mukobeko group of correctional facilities was obtained from the Commissioner General, Zambia Correctional Service (ZSC) headquarters. Formal written informed consent was administered to all inmates, and only those who consented were included in the study. All study data were restricted to the investigators, and no personal identifiers were used as data were being entered and analysed. All inmates who were found to be positive for intestinal parasites were referred to the nearest health facilities for standard care and management.

## CRediT authorship contribution statement

**Gilbert Munsaka:** Writing – original draft, Visualization, Validation, Supervision, Software, Resources, Project administration, Methodology, Investigation, Funding acquisition, Formal analysis, Data curation, Conceptualization. **Jonathan Gwasupika:** Writing – original draft, Visualization, Validation, Methodology, Investigation, Funding acquisition, Formal analysis, Data curation. **Ruth L. Mfune:** Writing – review & editing, Writing – original draft, Visualization, Validation, Methodology. **Imukusi Mutanekelwa:** Writing – original draft, Validation, Supervision, Software, Methodology, Investigation, Funding acquisition, Formal analysis, Data curation. **Bupe Chewe:** Supervision, Software, Resources, Project administration, Methodology, Investigation, Formal analysis, Data curation, Conceptualization. **Clyde M. Hakayuwa:** Writing – original draft, Visualization, Validation, Methodology, Investigation, Formal analysis, Data curation. **Mabvuto Daka:** Writing – review & editing, Writing – original draft, Validation, Software. **Mwape Chipampe:** Writing – original draft, Visualization, Validation. **Maisa Kasanga:** Writing – review & editing, Writing – original draft, Visualization, Validation. **Andrew M. Phiri:** Writing – original draft, Visualization, Validation, Supervision, Methodology. **Mable Mutengo:** Writing – review & editing, Writing – original draft, Visualization. **Oliver Mwenechanya:** Writing – review & editing. **Steward Mudenda:** Writing – review & editing, Writing – original draft, Visualization, Validation, Formal analysis, Data curation. **Victor Daka:** Writing – review & editing, Writing – original draft, Supervision, Software, Resources, Project administration, Methodology, Investigation, Funding acquisition, Formal analysis.

## Funding declaration

This research work received no specific grant from any funding agency in the public, commercial, or not-for-profit sectors.

## Declaration of competing interest

The authors declare that they have no competing interests.

## Data Availability

The datasets generated and/or analysed during the current study are available from the corresponding author on reasonable request.
