# Supplementary material for: Prevalence of intestinal parasites and associated risk factors among inmates at correctional facilities in Central Zambia
Source: Parasite Epidemiol Control. 2026 Apr 1;33:e00503. doi: 10.1016/j.parepi.2026.e00503 (PMC13084713; doi:10.1016/j.parepi.2026.e00503)
Supplement: Supplementary file 1 — Supplementary material [file mmc1.pdf]

**LMMU** Levy Mwanawasa Medical University

Let No One Be Left Behind

## RESEARCH ETHICS COMMITTEE

Telephone: +260-211-283827
Telegram:
Telex:
Telefax: +260-211-283827
IORG No. IORG0010491

Chainama Area
P.O. Box 33991
Lusaka, Zambia
Email: lmmurec@lmmu.ac.zm
OMB No. 0990-0279

**12th July, 2024**

**BUPE CHEWE.**
Levy Mwanawasa Medical University,
P.O. Box 33991,
**LUSAKA- ZAMBIA**

Dear Chewe,

**RE: PREVALENCE OF INTESTINAL PARASITES AND THE ASSOCIATED RISK FACTORS AMONG MUKOBEKO INMATES IN KABWE, ZAMBIA**

The above-mentioned research proposal that you submitted to the Levy Mwanawasa Medical University Research Ethics Committee (LMMU-REC) on 31th May, 2024.

Your proposal was approved based on the following documents:

1. The research protocol you submitted
2. Information sheets and informed consent
3. Data collection tools

**APPROVAL NUMBER: REF. No. LMMU-REC 0000464/24**

This number should be used on all correspondence, consent forms and documents as appropriate.

- **APPROVAL DATE: 11th July, 2024**

- **TYPE OF APPROVAL: Ordinary**

- **EXPIRATION DATE OF APPROVAL: 11th July, 2025**

After this date, this project may only continue upon renewal. For purposes of renewal, a progress report on a standard form obtainable from the LMMU-REC Offices should be submitted one month before the expiration date for continuing review.

❖ **SERIOUS ADVERSE EVENT (SAE) REPORTING:**

All SAEs and any other
serious challenges/problems having to do with participant welfare, participant safety and study integrity must be reported to LMMU-REC within 3 working days using standard forms obtainable from LMMU-REC.

✞ **MODIFICATIONS:** Prior LMMU-REC approval using standard forms obtainable from the LMMU-REC Offices is required before implementing any changes in the Protocol (including changes in the consent documents).

✞ **TERMINATION OF STUDY:** On termination of a study, a report must be submitted to the LMMU-REC using standard forms obtainable from the LMMU-REC Offices.

✞ **QUESTIONS:** Please contact the LMMU-REC:
Telephone No. at +260-211283827
email: lmmurec@lmmu.ac.zm.

✞ **OTHER:** Please be reminded to send in copies of your research findings/results for our records. You're also required to submit electronic copies of your publications in peer-reviewed journals that may emanate from this study. Use the LMMU online portal for further submissions.

**Congratulations!**

Yours sincerely,

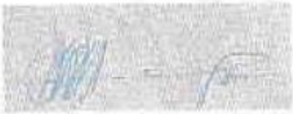

Dr Jimmy M. Hangoma
**ACTING VICE CHAIRPERSON**
Tel: 097 7534813
E-mail: jimmyhangoma0282@gmail.com
